# Patient's Information Needs and Understanding When Reading Anatomic Pathology Reports in Patient Portals

**DOI:** 10.1055/a-2731-4730

**Published:** 2025-11-17

**Authors:** Pavithra I. Dissanayake, Jacqueline Moss, Eta S. Berner, James J. Cimino

**Affiliations:** 1 The Edward Via Collage of Osteopathic Medicine–Virginia Campus (VCOM-VC), Virginia, United States; 2 School of Nursing, University of Alabama at Birmingham, Alabama, United States; 3 School of Health Profession (Posthumous), University of Alabama at Birmingham, Alabama, United States; 4 Department of Biomedical Informatics and Data Science, Heersink School of Medicine, The University of Alabama at Birmingham, Alabama, United States

**Keywords:** clinical documentation and communications, knowledge modeling and representation, pathology, patient portal, ontology

## Abstract

**Objective:**

To improve our understanding of the information needs of patients/caregivers when accessing anatomic pathology reports (APRs) via the patient portals (PPs) without further clarification by a clinician and to evaluate their opinion toward accessing APRs through a PP.

**Materials and Methods:**

We conducted an exploratory mixed-method study using a questionnaire and a think-aloud protocol based on three mock APRs of gastrointestinal specimens. Participants verbalized the cognitive processes used to understand report content, discussed concerns/questions, underlined all unknown terms/phrases, and summarized each APR in their own words. Interviews were transcribed and thematic analysis was performed. APR summaries and term descriptions were assessed for accuracy. Finally, the underlined terms/phrases were grouped to create an ontology.

**Results:**

All participants ( *n* = 15) had difficulty with most of the medical and technical terms in the APRs. Of the terms that participants said they were confident of knowing, only 48% were accurate and accuracy declined to 38% when guessed. The thematic analysis identified six main themes: information sources, interpreting the APR, emotional reactions, finding answers, opinion on accessing the APR on the portal, and preference for doctor–patient communication. The underlined terms were categorized into 12 domains creating the ontology of unknown terms in APRs.

**Conclusion:**

This study's findings suggest that patients, regardless of the diagnosis, have difficulties in understanding APRs, mainly due to complex medical terminology. Providing access to APRs via PPs alone is not sufficient to improve patient understanding. Further research is necessary to develop effective solutions that improve patients' understanding of these documents.

## Background and Significance

Patient engagement is integral for effective healthcare delivery. 1 It is widely accepted that patients who are actively engaged in their healthcare are more likely to follow their treatment plans, adhere to preventive behavior, be more engaged in medical decision-making, and have improved overall health outcomes. 2 3 4 5 6 7 8 Subsequently, literature has pointed to an increasing demand by patients for direct access to their medical records. 9 10 Several government policies, including the Information Blocking statute which is part of the 21st Century Cures Act Final Rule, have been implemented that mandate the immediate accessibility of electronic health information (EHI) to patients. 11 12 As a result, the health care industry is swiftly moving toward providing patients full access to their EHI through patient portals (PPs). 13

PPs are web-based patient-facing interfaces that allow patients and their caregivers 14 15 (e.g., parents of pediatric patients, family members of critically ill patients) access to patient's health data. The Health Information Technology for Economic and Clinical Health (HITECH) Act requires healthcare institutions to demonstrate the implementation and Meaningful Use of electronic health records (EHRs), including providing access to patient data through PPs. 11 16 17 Consequently, prior to the Information Blocking statute, institutions elected to release select data such as laboratory results, clinical notes, and complex medical reports at different time intervals ranging from hours to 28 days depending on the complexity of the report. 18

Anatomic pathology reports (APRs) are exemplars of complex medical documents written in specialized medical terminology that are now available in PPs. They are generated when patients undergo procedures leading to the extraction of tissue to provide diagnostic information. APRs are written by pathologists with specialized physicians as the audience to provide specific information that is crucial to create treatment and/or management plans. Therefore, APRs tend to have a highly specialized language that is unique to medicine and are inundated with highly technical jargon and medical terminology. Hence, even patients with adequate literacy may have difficulty understanding these complex medical documents. 19

The National Library of Medicine defines health literacy as “the degree to which individuals have the capacity to obtain, process, and understand basic health information and services needed to make appropriate health decisions.” 20 In the United States, the literacy of 54% of the adults is below sixth grade level, while 21% adults are considered illiterate in 2024. 21 Moreover, almost 9 out of 10 adults are thought to struggle with health literacy. 20 This underlines a significant issue regarding a patient's ability to understand complex medical reports such as APRs. Without the clarification provided by a clinician, patients may become confused and may easily misinterpret the diagnosis, possibly leading to unnecessary anxiety and impaired decision-making. Consequently, several studies have raised concerns regarding patients' ability to accurately understand complex medical documents. 18 22 23 24

Following the mandate of immediate release several studies have been conducted to assess patients' comprehensibility of radiology and APRs mostly centered around malignancies, especially with regards to APRs. 13 18 24 25 26 27 These studies have found that most patients have difficulty understanding the diagnosis as well as raised concerns over possible anxiety due to reports especially when the results are abnormal. Given these findings, it is likely that simply releasing these APRs via the PPs may not guarantee comprehension and automatically lead to improved patient involvement and health outcomes. In fact, some literature argues that new diagnoses received via PPs, especially related to cancer, may have negative impacts. 24 28 29

It is critical that we further investigate the impact of patients/caregivers accessing APRs through the PPs and identify specific difficulties associated with reading APRs without the input of a clinician. This will help better understand and create ways to improve comprehensibility. Prior studies on this subject have mostly used self-reported methodologies and/or concentrated on comparing the opinion of patients and clinicians related to release of medical reports in PPs. 13 27 28 They focused either on cancer diagnoses and/or specific terminologies and sections of the report. 24 25 26 27 28 29 30 In this study, we conducted in-depth interviews of patients reviewing both non-malignant and malignant APRs. We aimed to expand the literature by gaining insight into specific information needs related to accessing APRs through the PPs.

## Objectives

The objective of this study was to understand the information needs of patients/caregivers when accessing APRs without a clinician's interpretation similar to when accessed through PPs and their opinion toward receiving the APRs via PPs. The findings include a qualitative inductive thematic analysis, quantitative analysis of the accuracy of understanding of the APR content, and an ontological representation of the difficult-to-comprehend terms/phrases. Based on prior publications, we considered understandability to be a major cause of anxiety for patients associated with pathology data released via PPs. 22 23 24 28 29 Hence, we opted to design the study to identify specific information needs patients have and terms/phrases that patients find difficult to understand. We believe that the findings of this study will provide the basis to better understand patients' perception and difficulties faced by patients/caregivers when reading APRs on their own. It will also provide background information to possibly create tools to improve APR comprehension.

## Materials and Methods

### Study Design, Setting, and Recruitment

We employed a mixed-methods study design to answer the research questions which are listed in Table 1 . 30 31 A total of 15 participants were recruited from an endoscopy clinic waiting room at a large academic medical center in the Southeastern United States. As the primary goal was to determine the information needs of patients/caregivers when reviewing APRs via PPs, we considered any individuals who were 18 years of age or older, could speak and read English, and were neither an employee nor a student of the institution, as eligible to participate in the study. These were not direct patients receiving treatment at the time of the interview. Population-wide colon cancer screening is recommended via colonoscopy, which may result in biopsy and an APR. Hence, these individuals were deemed as appropriate subjects for this study. The study was approved by the Institutional Review Board of the University of Alabama at Birmingham.

**Table 1:** Research questions

Question	
1	What are patients' perceptions of having access to APRs via the PP?
2	How do patients currently attempt to understand the APR?
3	What questions arise when viewing an APR?
4	Are there specific concepts and domains that cause difficulty in understanding?
5	What may help improve the patient's understanding of the APR?

Abbreviations: APR, anatomic pathology report; PP, patient portal.

### Data Collection

Data were collected through individual semi-structured interviews with a questionnaire and a think-aloud protocol using mock APR-1, APR-2, and APR-3 containing benign, premalignant, and malignant diagnosis of gastrointestinal specimens, respectively ( Appendix contains the mock APRs). All mock APRs were created by a pathologist to simulate real-world APRs. All interviews were conducted in-person by one author (P.D.) and lasted approximately 30 to 40 minutes. The participants were given printed word documents of APRs in increasing severity of diagnosis by the interviewer once they completed the questionnaire. They were instructed to read the APRs out loud while verbalizing any difficulties that they encountered, the thinking process used to understand the difficulties, and any questions that arose. They were also asked to underline any terms and/or phrases that they did not understand or were unsure of. At the end of each APR, they were asked to summarize verbally the content in their own words. Participants were asked about their thoughts on what would help when viewing APRs via the PPs. Of note the study launched at the cusp of the coronavirus pandemic and hence, had to close early. A total of 15 interviews were conducted. They were audio-recorded and human transcribed.

### Quantitative and Qualitative Data Analysis

*Quantitative* : The demographic data from the questionnaires were aggregated. The patients' APR summaries were evaluated by a pathologist for the completeness and the accuracy of the diagnosis. The underlined terms/phrases were aggregated and grouped by terms the participants stated that they knew and those they guessed the meaning. These were then evaluated by a pathologist for accuracy and categorized as: accurate, inaccurate, partially accurate, and not interpreted. The frequency of each category was divided by the number of terms within the group to determine the frequency. Finally, the underlined terms/phrases were divided into concepts and domains to create the baseline ontology of difficult to understand terms.

*Qualitative* : An inductive thematic analysis approach was used to analyze the data from the think-aloud protocol. 32 33 The qualitative data analysis software NVivo was used to organize and code the data. 34 35 NVivo allows researchers to identify concepts within a text file and categorize and subcategorize them while maintaining the link to the original text. Multiple researchers can code the same text individually and overlap the data for comparison. The coding process involved the following: (1) all the authors (research team) coded the same first transcript; (2) the research team discussed the coding and arrived at a consensus for major codes and themes used in creating a codebook; (3) using the codebook, all team members individually coded a second same transcript that differed from the first; (4) new codes and coding discrepancies were discussed as a group. The interrater agreement was calculated (codes that not all coders agreed on during discussion were considered as disagreements). New agreed codes were added to the codebook; (5) remaining transcripts were coded by the lead author using the updated codebook; and (6) the team reviewed and finalized all the codes and themes.

## Results

### Participant Characteristics

In total, 15 participants were recruited for the study between January and March 2020. Many (40%) of the participants were between 61 and 75 years old and the majority (87%) had a bachelor's degree or higher. Table 2 summarizes the participant characteristics.

**Table 2:** Participant characteristics

Characteristic	Category		Frequency	Percentage
Participant number (n)			15	
Age	Less than 30		1	6.7
	30–45		4	26.6
	46–60		3	20.0
	61–75		6	40.0
	More than 76		1	6.7
Highest level of education	Less than high school degree		0	0.0
	High school degree		1	6.7
	Associate's degree		1	6.7
	Bachelor's degree		6	40.0
	Master's/Doctoral degree		7	46.6

### Questionnaire Responses

**Fig. 1:**
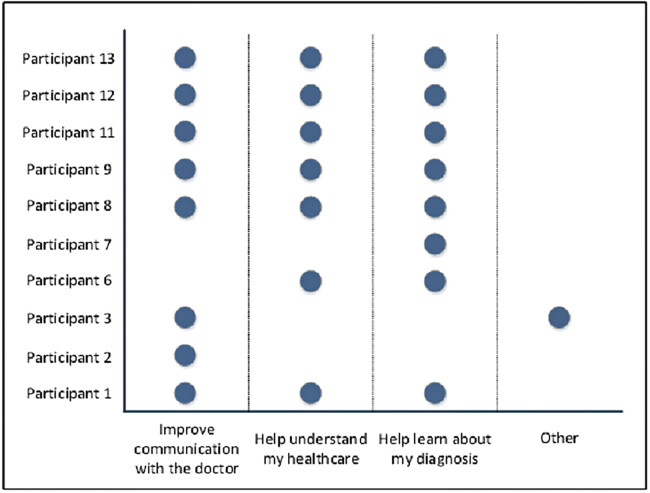
Reasons for accessing the anatomic pathology reports (APRs) in the patient portal (PP) ( *n* = 10; five participants indicated that they either did not use the PP or did not access APRs via the PP).

All participants currently relied on their physician for their personal medical information. The majority also used various online sources and family/friends, while four used other sources (e.g., books and PP). One participant only used their physician. Two-thirds of the participants wanted to see their APR through the PP before meeting with their physician, regardless of the diagnosis. Although, 60% had seen an APR prior to the study, only two participants had reviewed the APR before meeting with their doctor. Table 3 outlines the questionnaire responses. As shown in Fig. 1 , most participants picked multiple reasons for accessing APRs in the PP. One participant simply indicated wanting access to records as the reason which was listed under “other.”

**Table 3:** Participants' opinions and approaches to obtaining their medical information

	Category	Frequency	Percentage
Participant number (n)		15	
Current sources of health information	Doctor	15	100.0
	Family/friends	10	66.7
	Online sites	13	86.7
	Search engines (Google/Bing/Yahoo…)	12/13	92.0
	Health sites (WebMD, Medline plus)	9/13	70.0
	Professional sites (PubMed, UpToDate)	3/13	23.0
	Other	4	26.7
Willing to review APR in patient portal before conversing with the doctor	Yes, regardless of diagnosis	10	67.0
	Only if diagnosis is not serious	1	6.7
	No	4	27.0
Seen an APR before the study	Yes	9	60.0
	If yes, was it before doctor's appointment	2/9	13.0
Sources for answers to questions due to APR	Call my doctor	12	80.0
	Ask my family/friend	4	27.0
	Search online	7	47.0
Use patient portal		11	73.3

Abbreviation: APR, anatomic pathology report.

### Findings of the Thematic Analysis

*Interrater reliability:* Brennan and Silman interpreted a chi statistics value of ≥0.61 as a good measure of interrater reliability. 36 The average chi statistic value in our study was 0.72 (based on the codes of second transcript). The percentage agreement on individual codes across four coders ranged from 91 to 100% with an average percentage agreement of 98%.

The thematic analysis of the interviews discovered six main themes and multiple sub-themes nested within each theme. Four of the main themes directly addressed the five study questions: information sources, interpreting the APR, finding answers, and opinion on accessing the APR on the portal. Two additional main themes, emotional reactions and preference for doctor–patient communications that were indirectly related to the study focus, were also discovered. Table 4 lists the themes, sub-themes, definitions, and example quotes.

**Table 4:** Themes and subthemes along with their definitions and example quotes

*Theme*	*Sub-theme*	*Definition*	*Example quote*
Information sources	Personal healthcare provider	Source of medical information is their personal healthcare provider	“Unless you understand what you are reading you don't know, and you are not going to believe until you get confirmation when you talk to the doctor”
	Non-personal healthcare provider	Source of medical information is a health care provider who is not their personal physician (i.e., a physician at workplace or friend/relative who is a health professional)	“I have one daughter that is a doctor. Now if she were around, I would use her”
	Online sources	Discussion related to the use of online sources (i.e., google, WebMD, Pubmed, etc.) for medical information and concerns related to online sources	“This is where I would jump on google”
	Patient portal (PP)	Indicate that the patient portal is used to access his/her medical information	“Yes, it's [patient portal] a wonderful thing”
	Family and friends	Indicate that family members and/or friends who are not healthcare professionals assist with obtaining medical information	“My wife will be reading it over my shoulder… and find answers for me”
Interpreting the APR	Cognitive process used to understand	Cognitive process followed by the participants when reviewing the APR to understand the terms and/or phrases	“Immunohistochemical stains ok…. Umm… immune the is immune system. Histo is… I think it's like antihistamines. Umm and chemicals. But put it together and I'm not sure”
	Importance of sections of APR	Discussion of important and unimportant sections within the APR (i.e., diagnosis, gross description, all sections)	“They [sections of the APR] are all important”
	Reference to tissue size	Comments about the measurements within the APR	“I don't know centimeters and most people don't”
	Reason for difficulty in understanding	Capture reasons for the inability to understand the content within the APR (i.e., lack of medical training, unable to relate, unfamiliar terms, etc.)	“I don't know where that is cause you know, not a doctor”
	Unclear about the purpose or concept of APR	Participants are unclear of what leads to the generation of APR, when in the clinical workflow it is generated, and the finalized nature of the reported diagnosis (unless specifically stated otherwise) within the APR	“They [clinician] need to take it [specimen] to the lab and check it [specimen] to see”
	Vocabulary confident	Participant's self-reported confidence at knowing the meaning of a term/phrase within the APR	“I understand that it was an inflamed polyp”; “I don't know what that means”; “I assume that's some kind of….”
Emotional reactions	Relief	Description of feeling relieved and the reasons for the emotion (i.e., knowledge of diagnosis, benign diagnosis) in response to seeing the APR	“If you know what something is, even if you can't do anything about it, it reduces your apprehension and nervousness and fear”
	Stress	Description of feeling of stress (i.e., scare, panic, anxiety, etc.) and the reasons (i.e., long concern pause, nervous laugh, panic look) for the stress (i.e., cancer diagnosis) in response to seeing the APR	“Seeing all that I would be panicking”; “some people…. like my mom …. can't get online and look that up so for her to see that and not knowing she'll be nervous”
Finding answers	Questions	Participants state the questions that arose as they reviewed the APRs	“What are all these words that I have underlined mean?”
	Reasons for online search	Indicate reasons for searching online for answers to the questions	“I would… probably research all of these technical terms to make sure that I understood”
	Suggestions to improve understanding	Captures the suggestions made by the participants on how to improve the patient's understanding of the APR	“I would feel better, if it [APR] had definitions. And I can feel more like I understood what it said”
Opinion on accessing the APR on portal	Reasons for willingness to see the APR	Discussion of reasons/benefits of having the ability to access their APR through the patient portal prior to the doctor's appointment	“I would want to look at all these so when I have a conversation with the doctor it saves me time, it saves him or her time, and we can get on with the whole treatment”
	Reasons for unwillingness to see the APR	Capture reasons for not wanting to access their APR through the patient portal prior to their doctor's appointment	“Usually it [APR] is pretty technical, and I will have lot of questions. So why should I be frustrated”
	Considering other's opinion	Reflection of other peoples' preferences for having access to the APRs through the patient portal before the doctor's appointment	“Most people would like to see it [APR]”
	Change of opinion	Capture reasons for changing from willing to see the APR to not willing to see the APR prior to meeting with the doctor	“No, I don't think I would want to have it [APR]”
Preference for doctor–patient communication	Doctor call	Preference to have the doctor explain the APR either before or at the same time as the report is released in the patient portal or within a specific time from the report release	“I think in the face of a cancer diagnosis, phone call first, then send the report saying ‘please go over this before our meeting and make a list of questions’”
	No preference	No preference as to reviewing the APR with the doctor or in the patient portal	“I think I am impartial either way”

Abbreviation: APR, anatomic pathology report.

An important finding was that participants used many different cognitive techniques to decipher the APR content, some successfully and some unsuccessfully. Unsurprisingly, participants were familiar with words that are part of the layman's vernacular, like “carcinoma” and “colon.” When possible, participants used previous knowledge from prior health experiences and tried to guess the meaning of the terms by finding a close cognate of the unknown term/phrase or by breaking the word into parts.

Participants had many questions after reading the APRs mostly related to the meaning of the medical and technical terms. Some participants were unclear of the diagnosis and questioned “what are they finding?” They also found the diagnosis ambiguous when the clinical information (usually written by the clinician and/or more likely to contain lay terms) and diagnosis (written by the pathologist and contain medical terminology) had different disease names as in the case of APR-1. A few participants stated that they did not understand enough to even formulate questions.

It became evident that some participants were unaware that the APRs were created by the pathologist after the laboratory processed the specimens to provide results/diagnoses. When asked to restate the diagnosis after reading the APR, several participants repeatedly stated how they would wait for the results to know the diagnosis. It was evident that participants, when faced with the inability to clearly understand the diagnosis, would revert to believing that the reported diagnosis was the initial examination, and additional findings would be reported later.

**Fig. 2:**
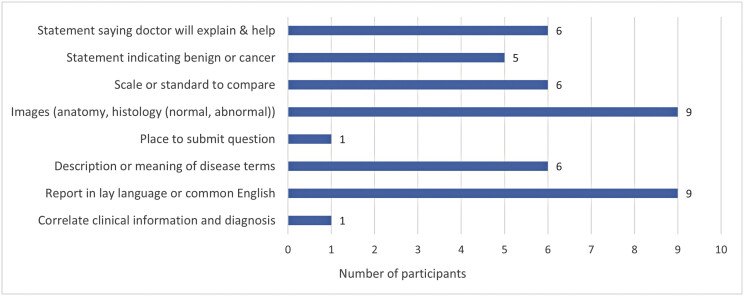
Suggestions to improve anatomic pathology report comprehensibility.

Participants offered valuable suggestions for improving APRs ( Fig. 2 ). The most common recommendations were to include visuals, either of histology or general gastrointestinal anatomy, and to use common English terminology in the report. Many also suggested defining medical terms, offering standard for comparison, and including a statement reassuring that the physician will explain the APR.

### Accuracy of Term Interpretation and APR Summary

**Fig. 3:**
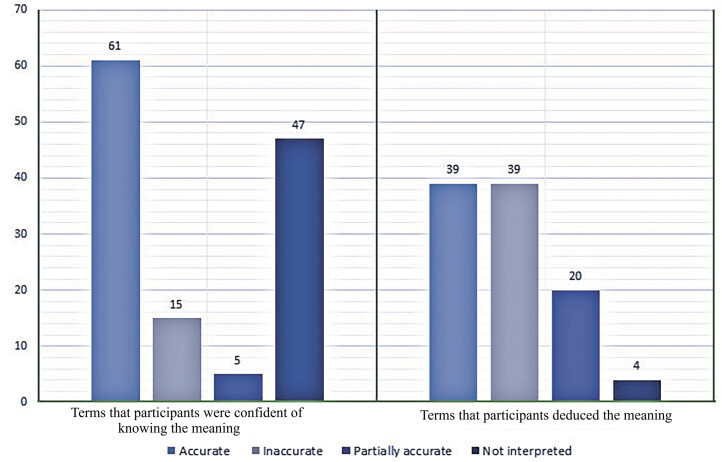
Accuracy of interpretation of terms/phrases within the anatomic pathology reports: Accurate = participants' interpretation was correct; Inaccurate = participants' interpretation was incorrect; Partially accurate = participants' interpretation was only partly correct; and Not interpreted = the participant did not provide an interpretation for the term. (The number above each column represents the total number of terms within each category aggregated across all participants.)

The descriptions given by participants for terms/phrases that they were either confident of knowing or presumed/deduced the meaning of were evaluated for accuracy, categorized based on level of accuracy, and are depicted in Fig. 3 . Participants were accurate in only 48% and partially accurate in 4% of the terms that they were confident of knowing. They did not provide a description for 47 (37%) terms that they claimed to know. Of the terms for which the meaning was guessed only 38% were accurate while 38% were inaccurate. Table 5 provides examples of terms/phrases that participants either confidently indicated that they knew accurately (categorized based on accuracy) or were uncertain of the meaning but defined accurately.

**Table 5:** Examples of interpreted terms/phrases based on participants' confidence and accuracy of the interpretation

*Category*	*Term/phrase*	*Quote*
Not confident of but interpreted accurately	Tumor cells	“Think cancer?”
	Rectosigmoid	“That's lower colon I guess”
	Adenocarcinoma	“A cancer I think”
	Villi	“That's the little hairlike things, maybe”
	Duodenum	“Part of the intestine”
Confident but interpreted inaccurately	Lymphocytosis	“Inflammation of the lymph nodes”
	Resection margins negative for cancer	“Tumor was non-cancerous”
	Intraepithelial (within the duodenum)	“Layer of skin”
	Small vessel lymphovascular invasion	“Muscle is involved and that a lymph nodes is involved”
	Polyp with granulation tissue	“That means not smooth”
Confident and interpreted with partial accuracy	Gluten-sensitive enteropathy	“Its not a disease but it is a gluten sensitive”
	Small vessel lymphovascular invasion is focally seen	“They can see that the cancer cells are going to other tissue”
	Lymphovascular invasion	“Lymph system”
	Resection margins are negative for cancer	“Some of it was cancerous but maybe outside of it wasn't”'
	Immunohistochemical staining	“They did some sort of immune rection”
Confident but the subject did not provide an interpretation	Inflamed hyperplastic polyp	“I know what they mean”
	Bisected	“I understand all that”
	Villous	“I know what that means”
	Gluten sensitive	“I understand”
	Crypt	“I know what that means”

**Fig. 4:**
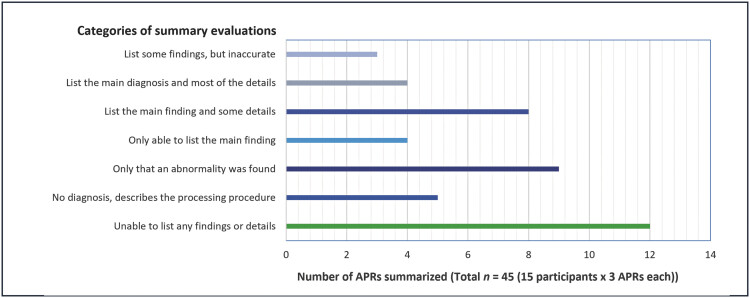
Ability of the participants to summarize the anatomic pathology reports (APRs).

Fig. 4 illustrates the findings from the evaluation of participants' summaries of the APRs, as assessed by a pathologist for accuracy. Most participants recognized “carcinoma” as cancer in APR-3, which accounts for majority of the responses within “main finding” category. In contrast, many participants found non-malignant APRs (APR-1, APR-2) more challenging, resulting in more responses classified as “unable to list any findings or details” or “only that an abnormality was found.” In three cases, the summaries were deemed inaccurate by the pathologist. These findings highlight that participants were more confident in identifying malignant findings than benign ones. This suggests a potential gap in identifying non-malignant diagnoses.

### Ontology of Unknown Terms in APRs

**Fig. 5:**
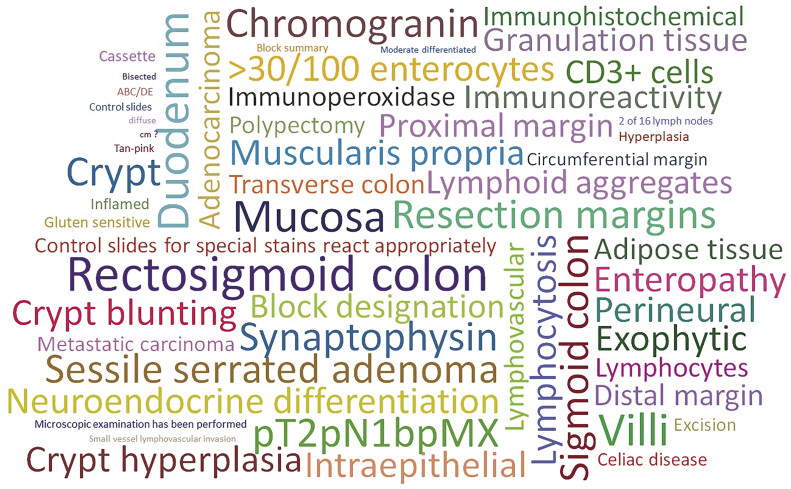
Word cloud of unknown terms and phrases.

Analysis of the underlined terms/phrases revealed that most participants had difficulty with terms in anatomy, histology, and pathology (e.g., chromogranin, pT2pN1bMX, enterocytes) domains. The most underlined terms were “rectosigmoid colon” followed by “villi,” while only a few participants underlined technical terms such as cassette, bisected, and block summary. Fig. 5 depicts the frequency of these terms.

**Fig. 6:**
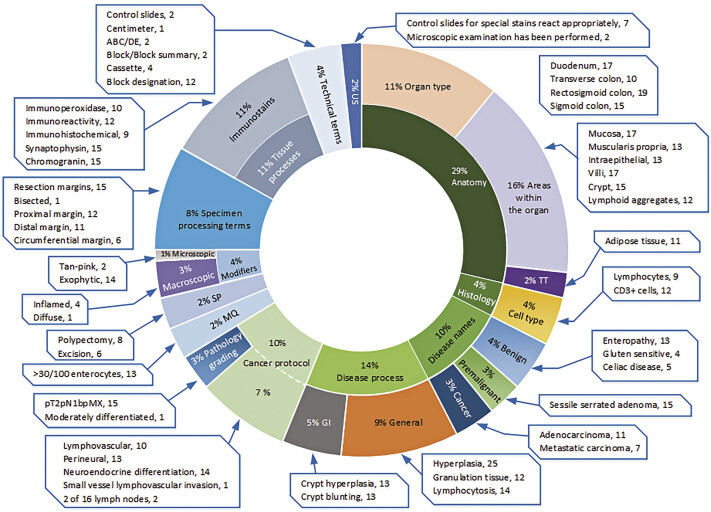
Ontology of unknown terms in anatomic pathology reports: inner ring represents the domains, the numbers next to concepts (e.g., Enteropathy, 13) represent the number of times that term was identified by participants as unknown, and the percentages within the rims represent the proportion of each category.

We classified these terms based on different concept groups as an attempt to identify the generalized categories for unknown terms. A total of 12 domains were identified. Five domains did not have children. Paralleling the word cloud, we found that the largest (29%) domain with difficulty was *anatomy* , which contained the concepts *organ type* , *areas within the organs* , and *tissue types* . *Disease process,* which was defined as abnormal histology changes associated with the body, was the second largest domain followed by *tissue processes* . The ontology's semantic relationships were limited to simple parent–child (“ *is_a* ”) relationships between concepts. Fig. 6 depicts the framework of the ontology for grouping underlined terms. Table 6 lists domains, concepts, and definitions.

**Table 6:** Ontology of unknown terms and phrases

*Domain*	*Concepts*	*Definition*
Anatomy		Names of body structures
	Organ type	Names of organs
	Areas within the organ	Location of the structures within organs
	Tissue type	Names of tissue types
Histology		Names of microscopic anatomy of human body
	Cell type	Names of cell types
Disease names		Names of disease
	Benign	Names of diseases that are benign
	Premalignant	Names of diseases that are pre-cancerous
	Cancer	Names of diseases that are cancer
Disease process		Abnormal histological changes that are associated with the body
	General	Names of abnormal histological changes that can be seen anywhere in the body, not limited to one organ (i.e., lymphocytosis, granulation tissue)
	Gastrointestinal	Names of abnormal histological changes that are unique to gastrointestinal system
Cancer protocol		Terms used by pathologist to describe information related to cancer diagnosis and are found with cancer protocols
	Pathology grading	Grading systems for severity of pathologies, malignant and non-malignant
Microscopic quantifications		Listing of quantities of microscopic examination
Surgical procedures		Names of surgical procedures
MODIFIERS		Adjectives used to describe the tissue
	Macroscopic	Adjectives describing the microscopic characteristics of a tissue
	Microscopic	Adjectives describing the macroscopic characteristics of a tissue
Specimen processing terms		Description of specimen processing processes performed within the pathology lab to make histology slides for examination
Tissue processes		Specimen processing method performed following initial pathology diagnosis (i.e., immunostains, molecular, flow cytometry)
	Immunostains	Names of immunostains used to highlight antigens within the tissue
Technical terms		Technical terms used in the APR (i.e., control slides, cm, etc.)
Unclear sentences		Unique sentences regularly included in the APRs that patients find confusing

## Discussion

In this study, we investigated participants' ability to understand APRs without the assistance of a clinician and found that participants had considerable difficulty in accurately understanding the report in its current format. The link between poor health literacy, defined as readability and comprehensibility of the medical documents, and adverse health outcomes is well known. 37 38 39 Even though there have been few studies conducted attempting to illuminate the perceptions, emotional burden, and comprehensibility associated with receiving complex medical reports through the PPs, to our knowledge these studies either followed a self-reported survey methodology, compared the clinician versus patient perception of obtaining results via PPs, and/or only looked at reports containing malignancy diagnoses. 18 22 23 27 40 41

Recently, a few studies have looked at patients' experiences and challenges related to APRs with cancer diagnoses. Austin et al studied patients' perspectives related to APRs with breast and colorectal cancers using focus groups and deduced that the complex language and format of the APRs can lead to confusion and uncertainty. 23 Our study, in addition to confirming their findings, expanded on specific types of terms a lay person would have difficulty understanding and types of associated information needs both related to benign and malignant APRs by looking at multiple APRs as a whole.

Our study yielded a surprising revelation regarding the types of words that posed the greatest challenge to participants. Although terms describing basic anatomy, such as “colon” and “intestine,” are generally considered common knowledge, we discovered that when these familiar terms were paired with specific anatomical descriptors like “duodenum” or “sigmoid,” participants struggled to specify the location. For example, “rectosigmoid colon” is the most common unknown term. This finding highlights a notable gap between recognizing basic anatomical terms and comprehending more precise medical vocabulary, even when it incorporates familiar components. In 2022, a survey conducted by Verosky at al discovered that patients were able to differentiate between benign and malignant in most cases when reviewing breast pathology terminology, though they rarely were able to define carcinoma accurately. 24 In contrast, our study noted carcinoma to be a familiar term. It is possible that this difference stems from the difference in evaluation methods. Verosky study asked participants to define eight specific terms related to breast pathology in isolation, while we asked participants to interpret full pathology reports without pointing to specific terms. Additionally, a significant number of subjects in Verosky study were in medical field/health care worker, undergoing cancer treatment or surveillance at the time of the survey, and/or had a previous tissue biopsy. The participants of our study were not undergoing cancer treatment/surveillance. This difference in patient population also likely contributed to the difference in knowledge between these studies further iterating the importance of studying the population directly due to marked variability of patient characteristics and subject content within APRs. It is evident that more detailed and broad studies are needed to better understand these fine yet important information gaps among different patient populations.

An important unexpected finding of our study is that in some cases participants were unclear about the purpose of an APR, particularly when faced with unfamiliar diagnostic terms. This uncertainty often led to increased anxiety. The context of receiving a pathology report, which typically follows the removal of tissue due to an abnormality, inherently creates a stressful environment due to the possibility of unfavorable news. Participants stated that they were relieved when they understood the diagnostic terms, whether benign or malignant. As one participant indicated, it is a relief to know even if it is cancer. However, when the diagnosis was unclear due to incomprehensible terminology, participants showed increased anxiety due to the uncertainty. Notably, these difficulties with APRs were not limited to cancer diagnoses. As one participant indicated, “it is a relief to know even if it is cancer.” Additionally, unfamiliarity with the APR layout contributed to confusion. For example, when questioned about the diagnosis after the think-aloud protocol, the participants often looked for the diagnosis at the end of the document rather than at the beginning. Although there are Web sites describing how to read APRs in general, it may be beneficial for the clinician to emphasize the APR's purpose prior to the procedure or post a description with the APR in the PP. 42 To our knowledge, this phenomenon has not been discussed in existing literature and should be studied further, especially when APRs are released in PPs.

It is generally accepted that patients want access to their medical records, and that having patients involved in their healthcare will lead to better health outcomes. 1 2 3 4 43 44 Although we found this to be true, we also discovered that a few participants in our study prefer to communicate with their doctor prior to seeing the APR in the PP. They reasoned that incomprehensible terminology would prompt uncertainty and more questions leading to increased anxiety. Interestingly, we also noted that many participants who initially wanted access to APRs via PP regardless of the diagnosis, changed their mind when faced with a cancer diagnosis. Given these findings, perhaps accessibility for APRs on PPs prior to meeting with a clinician should be based on individual preferences following an explanation of all possibilities. Further investigations are needed to better understand when and what APRs to release via PPs to prevent harm.

Based on existing literature, patients use online resources the most even though they trusted and relied on their physicians more. 42 44 45 46 Our study participants also relied on their doctor; however, unlike previous reports, our participants turned to family and friends more than online sources. Participants indicated trustworthiness and information overload as reasons for apprehension toward Internet sources, whereas family and friends, especially those in healthcare, regardless of their field and training, were seen as being able to decipher the APRs without overwhelming additional information.

Prior studies aimed at improving patients' APR comprehensibility have focused on either creating direct communications between the pathologists and patients, limited to cancer reports, or are describing tools that are not directly connected to APRs. 23 47 48 49 Another approach discussed in the literature is a patient-centered redesigning of medical documentation. 23 49 50 These implementations, though immensely valuable, require additional workflows by pathologists or depend on patients accessing additional tools. By contrast, we attempted to identify specific terminology and information needs from the patient's perspective. As depicted in Fig. 2 , participants had many suggestions on ways to improve APR readability. Moreover, we believe that identifying complex terminology based on patients' input and creating an ontology using those terms, similar to Fig. 6 ( Table 5 ), are essential in creating patient-centered APRs.

Patients may employ various cognitive techniques to comprehend complex medical literature. One key approach is cognitive restructuring, which involves identifying and challenging unhelpful assumptions about medical information, replacing them with more balanced interpretations. 51 Problem-solving skills are also utilized to break down complex medical concepts into more manageable parts, facilitating better comprehension. 51 Developing a greater sense of self-efficacy in one's ability to understand medical information may boost confidence and motivation to engage with literature. 52 Research exploring patients' strategies for interpreting unfamiliar medical information, particularly within APRs, is scarce. Current studies have not adequately addressed the cognitive techniques and resources patients employ for comprehension of complex medical data. This gap in literature limits our understanding of how patients navigate and make sense of challenging medical concepts, potentially restricting our ability to create helpful tools to assist patients.

Our study is limited by the number of participants and the use of only three APRs. Given the exploratory nature of the study, it is also possible that some areas of need are not fully represented by the results such as the emotional burden and steps taken to clarify unknown terms found within the reports. The fact that the participants are not real patients viewing their own real APRs may also influence the findings. However, we believe that the difficulties and the information needs identified are generalizable as they are not unique to a specific population. Thus, the study methodology is proven sound in identifying specific concepts and questions encountered by patients related to APRs and adds valuable insights to fill gaps within the existing knowledge that could be used to assist patients.

In the future, we hope to conduct a larger similar study with a variety of APRs including different terms/phrases creating a more robust ontology. We believe such an ontology would be extremely helpful for researchers and program developers aiming to develop tools to improve APR comprehensibility. Observational studies involving real patients accessing APRs via PP along with access to online resources and clinicians, as well as creating and testing tools to help patients is another great direction for future research. A study investigating specific cognitive techniques used by patients to decipher unknown information, prior to developing support tools, will greatly assist in determining the best types of tools to help patients. Additionally, this study's findings pique the curiosity regarding information needs of non-pathology physicians related to APRs, hence would be another stimulating avenue for future research. As a rule, healthcare organizations should be mindful of how patients interpret the data presented within the PPs, the information needs related to these reports, and the sources that the patients rely on for answers.

## Conclusion

The present study examined patients' comprehension of APRs when presented without additional assistance, simulating their release through PPs. Our findings revealed that participants encountered significant challenges in understanding APRs, primarily due to unfamiliar terminology and would benefit from having a tool to assist with complex medical terminology. Our results suggest that there is a need for targeted interventions to support patients in interpreting these complex medical documents. Additional research should be done to further understand patient needs and how best to aid patients' understanding with the ultimate goal on creating tools that improve patients' comprehension.

## Clinical Relevance Statement

Complex medical documentations such as APRs are inundated with medical and technical terminologies that are beyond the scope of a typical patient's vocabulary. Simply providing patients access to these reports as mandated by the government most likely will not lead to improved patient involvement in care and better patient outcomes due to comprehension difficulties experienced by most patients when accessing APRs via patient portals. Uncovering the information needs associated with APRs and incorporating tools to meet those needs can greatly assist patients better understand their APRs when accessed through patient portals ultimately leading to better patient outcomes.

## Multiple Choice Questions

1. When a patient reviews an anatomic pathology report via the patient portal prior to meeting with his/her clinician to provide background information related to the report/diagnosis, which of the following aspects of the report if present would likely cause the patient significant anxiety/stress?
  - Diagnosis that he/she knows is benign
  - Unknown anatomy terms
  - Previously known diagnosis
  - None of the above
  **Correct Answer:** The correct answer is option b. Anatomic pathology reports contain many specific detailed medical terms that indicate specific anatomic location of the specimen, histologic descriptions, diagnostic details, and many other technical terms. Most patients had difficulty understanding these terms and noted that inability to understand these terms which leads to confusion caused significant amount of stress and anxiety.
2. Which of the following modifications is most likely to assist patients better understand their APRs when accessed through the patient portal?
  - Including explanations for the medical terms written in the report
  - Formatting the APR to clarify the diagnosis
  - Rewriting the APR in common English language
  - All of the above
  **Correct Answer:** The correct answer is option d. Patients found both the format and the language of the existing APRs, which are written with physicians as the audience, confusing. Incorporating tools to provide explanations to complex medical terms included in the report, reformatting the report so the diagnosis is easily identifiable, and/or including a second version of a report written in lay English language with non-medical personnel in mind, will all improve patients' comprehensibility of APRs.
