# Supplementary material for: Patient's Information Needs and Understanding When Reading Anatomic Pathology Reports in Patient Portals
Source: ACI open. 2025 Nov 17;9(2):e65–78. doi: 10.1055/a-2731-4730 (PMC13393550; doi:10.1055/a-2731-4730)
Supplement: Supplementary file 1 — Supplementary Material [file 10-1055-a-2731-4730_27486303.pdf]

Appendix

The mock anatomic pathology reports (APR) used for the study: APR-1

Anatomic Pathology Laboratories, Inc
1000 Anatomic Rd
Pathology, AP 54321

**Patient Name:**	**Patient, J D**	**Case:**	**SP-21-0001**
Sex:		Location:	Central Lab
DOB:	01/01/1990	Collection Date:	01/01/2021
Med. Rec. #:	123456	Received Date:	01/01/2021
Physician(s):	Gi Doctor, MD	Reported Date:	01/03/2021

SURGICAL PATHOLOGY

DIAGNOSIS

Intestine, small, duodenum, biopsy:

- Mucosal lymphocytosis with variable villous abnormality and crypt hyperplasia, consistent with gluten sensitive enteropathy

Anatomy A. Pathologist, MD
(Electronically signed by)
Verified: 01/03/2021 01:12
ABC/DE

Clinical Information

The patient is 31 years old and is with symptoms suspicious of celiac disease

Gross Description

The specimen is received in a container labeled with the patient's name, medical record number and is designated "duodenum biopsy". It consists of a tan-pink fragment of soft-tissue measuring 0.3 x 0.3 x 0.3 cm. The specimen is entirely submitted in one cassette.

Dr. Anatomy/Dr. Pathologist
01/01/2021 10:10:10 CDT
ABC/DE

Block Summary

A1- Duodenum biopsy

Microscopic Description

ACI Open | DOI 10.1055/a-2731-4730 | © The Author(s).

Diffuse increase in intraepithelial lymphocytes along the villi are seen with an increased number of CD3+ cells (>30/100 enterocytes). Scattered lymphoid aggregates are also seen. Immunoperoxidase stains show crypt hyperplasia. Focal crypt blunting is seen. Control slides for the special stains react appropriately.

Dr. Pathologist
01/03/2021 10:10:10 CDT

The mock anatomic pathology reports (APR) used for the study: APR-2

Anatomic Pathology Laboratories, Inc
1000 Anatomic Rd
Pathology, AP 54321

**Patient Name:**	**Patient, J D**	**Case:**	**SP-21-0002**
Sex:		Location:	Central Lab
DOB:	01/01/1900	Collection Date:	01/01/2021
Med. Rec. #:	112233	Received Date:	01/01/2021
Physician(s):	Gi Doctor, MD	Reported Date:	01/03/2021

SURGICAL PATHOLOGY

DIAGNOSIS

A. Colon, transverse, polypectomy:
   - Sessile Serrated Adenoma

B. Colon, rectum, polypectomy:
   - Inflamed hyperplastic polyp with granulation tissue

Anatomy A. Pathologist, MD
(Electronically signed by)
Verified: 01/03/2021 01:12
ABC/DE

Clinical Information

Screening colonoscopy. The patient is 121 years old.

Gross Description

The specimen A is received in a container labeled with the patient's name, medical record number and is designated "transverse colon polyp". It consists of a tan-pink fragment of soft-tissue measuring 1.5 x 0.6 x 0.3 cm. Resection margin is inked black. The specimen is bisected and entirely submitted in one cassette.

The specimen B is received in a container labeled with the patient's name, medical record number and is designated "rectum polyp". It consists of a tan-pink fragment of soft-tissue

ACI Open | DOI 10.1055/a-2731-4730 | © The Author(s).

measuring 0.3 x 0.2 x 0.1 cm. Resection margin is inked black. The specimen is entirely submitted in one cassette.

Dr. Anatomy/Dr. Pathologist
01/01/2021 10:10:10 CDT

Block Summary
A1- Transverse colon polyp
B1- Rectal polyp

Microscopic Description
A microscopic examination has been performed.
Dr. Pathologist
01/03/2021 10:10:10 CDT
The mock anatomic pathology reports (APR) used for the study: APR-3

Anatomic Pathology Laboratories, Inc
1000 Anatomic Rd
Pathology, AP 54321

**Patient Name:**	**Patient, J D**	**Case:**	**SP-21-0003**
Sex:		Location:	Central Lab
DOB:	01/01/1950	Collection Date:	01/01/2021
Med. Rec. #:	123123	Received Date:	01/01/2021
Physician(s):	Gi Doctor, MD	Reported Date:	01/03/2021

SURGICAL PATHOLOGY

DIAGNOSIS

Colon, rectosigmoid, excision:

- Moderately differentiated adenocarcinoma with focal neuroendocrine differentiation in sigmoid colon
- Tumor size is 3.2 cm in greatest dimension
- Tumor invades into, but not through muscularis propria
- Small vessel lymphovascular invasion is focally seen
- No perineural invasion identified
- Resection margins are negative for cancer
- Two of sixteen lymph nodes are positive for metastatic carcinoma (2/16)
- Pathologist stage is pT2pN1bpMX
- See the attached cancer summary for complete details

Anatomy A. Pathologist, MD
(Electronically signed by)
Verified: 01/03/2021 01:12
ABC/DE

ACI Open | DOI 10.1055/a-2731-4730 | © The Author(s).

Clinical Information

A 71 year old patient with a sigmoid mass.

Gross Description

The specimen is received in a container labeled with the patient's name, medical record number and is designated "rectosigmoid colon". It consists of a segment of colon measuring 9.8 x 5.6 x 3 cm. The specimen is opened revealing an exophytic mass measuring 3.5 x 3 cm in in greatest dimension located 2.5 cm from the proximal margin and 3.8 cm from the distal margin. Dissection through the mass reveals a depth of 2.7 cm. The circumferential margin is 1.8 cm. The remaining mucosa is tan-pink and with normal folds. No other lesions are identified. The attached adipose tissue is dissected for lymph nodes and 16 possible lymph nodes are submitted. Representative sections are submitted as listed under block summary.

Dr. Anatomy/Dr. Pathologist
01/01/2021 10:10:10 CDT

Block Summary

A1	- Proximal margin
A2 - 3	- Distal margin
A4	- Normal mucosa
A5 - 8	- Mass
A9	- Circumferential margin
A10 - 15	- Possible lymph nodes

Microscopic Description

A microscopic examination has been performed.
Dr. Pathologist
01/03/2021 10:10:10 CDT

Special Stains

Immunohistochemical stains for synaptophysin and chromogranin show focal positive immunoreactivity in tumor cells. Control slides for the special stains react appropriately.

ACI Open | DOI 10.1055/a-2731-4730 | © The Author(s).
